# The Dose-Dependent Effects of Vascular Risk Factors on Dynamic Compensatory Neural Processes in Mild Cognitive Impairment

**DOI:** 10.3389/fnagi.2018.00131

**Published:** 2018-05-08

**Authors:** Haifeng Chen, Fan Su, Qing Ye, Zan Wang, Hao Shu, Feng Bai

**Affiliations:** Department of Neurology, Affiliated ZhongDa Hospital, School of Medicine, Southeast University, Nanjing, China

**Keywords:** vascular risk factors, mild cognitive impairment, default mode network, dose-dependent effects, compensation

## Abstract

**Background/Objectives:** Mild cognitive impairment (MCI) has been associated with risk for Alzheimer's Disease (AD). Previous investigations have suggested that vascular risk factors (VRFs) were associated with cognitive decline and AD pathogenesis, and the intervention of VRFs may be a possible way to prevent dementia. However, in MCI, little is known about the potential impacts of VRFs on neural networks and their neural substrates, which may be a neuroimaging biomarker of the disease progression.

**Methods:** 128 elderly Han Chinese participants (67 MCI subjects and 61 matched normal elderly) with or without VRFs (hypertension, diabetes mellitus, hypercholesterolemia, smoking and alcohol drinking) underwent the resting-state functional magnetic resonance imaging (fMRI) and neuropsychological tests. We obtained the default mode network (DMN) to identify alterations in MCI with the varying number of the VRF and analyzed the significant correlation with behavioral performance.

**Results:** The effects of VRF on the DMN were primarily in bilateral dorsolateral prefrontal cortex (DLPFC) (i.e., middle frontal gyrus). Normal elderly showed the gradually increased functional activity of DLPFC, while a fluctuant activation of DLPFC was displayed in MCI with the growing number of the VRF. Interestingly, the left DLPFC further displayed significantly dynamic correlation with executive function as the variation of VRF loading. Initial level of compensation was observed in normal aging and none-vascular risk factor (NVRF) MCI, while these compensatory neural processes were suppressed in One-VRF MCI and were subsequently re-aroused in Over-One-VRF MCI.

**Conclusions:** These findings suggested that the dose-dependent effects of VRF on DLPFC were highlighted in MCI, and the dynamic compensatory neural processes that fluctuated along with variations of VRF loading could be key role in the progression of MCI.

## Introduction

Alzheimer's Disease (AD) is the most common neurodegenerative disorder in elderly people worldwide. It encompasses the progressive continuum of impairment from mild cognitive impairment (MCI) whose potential pathophysiology is related to AD to dementia (Albert et al., 2011). There is growing evidence that vascular risk factors (VRFs) are associated with cognitive impairment and AD pathogenesis (Luchsinger et al., 2005; Bangen et al., 2015). The underlying effects of VRFs on neural networks and their inherent neural underpinnings in MCI remains relatively unknown.

Previous studies have suggested that AD has a complex and multifactorial etiology, including neurovascular hypothesis (i.e., neurovascular dysfunction). An enhanced understanding of this heterogeneous disorder pathogenesis and the neurovascular hypothesis have suggested that a cascade of consecutive changes originate from genetic risk factors associated with vascular damages (e.g., APOE ε4, PICALM, CLU, PSEN, and MEOX2) (Nelson et al., 2016), VRFs (e.g., hypertension, diabetes, hypercholesterolemia, smoking and alcohol abuse) (de Bruijn and Ikram, 2014; Nelson et al., 2016), and cerebrovascular diseases initiating the disintegration of the neurovascular unit mediated by multiple physiopathology pathways (Zlokovic, 2005; Bell and Zlokovic, 2009; Zenaro et al., 2017). In the light of progressive disequilibrium, cerebral hypoperfusion and impaired clearance of β-amyloid (Aβ) across the blood-brain barrier (BBB) have been major and interactive parts of the pathological cascade, which result in synaptic and neuronal dysfunction, injury, and loss in turn (Bell and Zlokovic, 2009; Nelson et al., 2016; Zenaro et al., 2017). Previous studies have demonstrated that VRFs also commonly contribute to vascular cognitive impairment and dementia (VCID) (Gorelick et al., 2011). It is generally recognized that there is interrelationship and difference between VCID and AD (Nucera and Hachinski, 2018). VRFs-related endothelial dysfunction, in turn leading to chronic cerebral hypoperfusion, may be a key pathophysiological mechanism of VCID (Duncombe et al., 2017). These VRFs have been known as modifiable risk factors currently. It is possible for at risk individuals to prevent or delay AD by addressing these factors. There has been abundant and compelling evidence showing its feasibility (Love and Miners, 2016; Maliszewska-Cyna et al., 2017).

The default mode network (DMN) is considered a constellation of collective brain regions with high degrees of functional connection that are activated during rest but deactivated during task performance (Raichle, 2015). The behavioral significance of the DMN has highlighted in many areas, including the consolidation of memory, the self-referential processing, and the monitoring of external and internal environments (Buckner et al., 2009). The DMN is considered to be a preferential (but not single) site for amyloid burden related to cortical atrophy and metabolic disturbance during the early phase of AD (Oh et al., 2014; Chang et al., 2015; Grothe and Teipel, 2016). Prior fMRI studies have shown that DMN dysfunction could be used as a noninvasive and distinctive biomarker to predict and monitor the progression of AD. A longitudinal study showed that a substantial decrease of PCC/precuneus functional connectivity occurred at the follow-up for amnestic-MCI (aMCI) subjects, compared to the control group (Bai et al., 2011). There is a progressive weakening trend of functional connections within the DMN that is prominent starting in the normal elderly going to aMCI and then AD (Zhu et al., 2013). Therefore, the specific patterns of the progressive alterations of the DMN could be of considerable value when identifying and monitoring the conversion to AD. When and if VRF has the typical effects on the DMN remains unanswered.

There has been a proliferation of DMN functional connection studies that explore the effects of single VRF in AD. There have been few studies that focus on multiple VRFs. The present study was performed with MCI individuals and cognitively normal elderly subjects with or without VRFs. The primary objective of this study was to identify significantly altered DMN caused by the interactive effects of VRFs and disease statement. The secondary aim was to examine if the functional connectivity in the interaction region was correlated with cognitive function. We explored the underlying trend of the functional connectivity in the interaction region from normal elderly to MCI with the increasing loads of VRF. We hypothesized that variations in the quantity of VRFs could result in remodeling the DMN and lead to an abnormal neural compensatory mechanism.

## Materials and methods

### Subjects

The present study employed 128 elderly Han Chinese participants (67 MCI subjects and 61 matched normal elderly) who were who were chosen from the memory clinic and communities according the following inclusion and exclusion criteria. It was approved by the Research Ethics Committee of Affiliated ZhongDa Hospital, Southeast University, and signed informed consent was obtained from all participants. The MCI subjects included in the study were diagnosed based on the recommendations of Petersen et al. and National Institute on Aging-Alzheimer's Association (NIA-AA)(Petersen, 2004; Albert et al., 2011): (i) subjective memory impairment corroborated by the subject and/or an informant; (ii) objective memory performance documented by an auditory verbal learning test-delayed recall (AVLT-DR) score less than or equal to 1.5 standard deviation (SD) of age-adjusted and education-adjusted norms; (iii) mini mental state examination (MMSE) score ≥ 24; (iv) clinical dementia rating (CDR) of 0.5; (v) no or minimal impairment in activities of daily living; and (vi) absence of dementia or insufficient dementia to meet the criteria of NIA-AA for AD. In addition, participants with regular use of drugs or medications, a history of other neurological or psychiatric illness (e.g., stroke, depression and others), and contraindications to MRI scanning were excluded in the present study. Besides, control subjects were required to have a Clinical Dementia Rating of 0, MMSE score ≥ 26, AVLT-DR > 4, and eight or more years of education. All these diagnostic processes were completed by an experienced neuropsychiatrist who administered structured interviews with the participants and their informants.

### VRF and subgroups

The VRF, including hypertension, diabetes mellitus, hypercholesterolemia, smoking and alcohol drinking, established by various previous researches were considered into this study (Reitz et al., 2007; de Bruijn and Ikram, 2014; Zhou et al., 2014). Medical records were collected to clinical assessment by an experienced neurologist adopting the questionnaire method. The assessment included presence of hypertension (systolic/diastolic blood pressure > 140/90 mmHg or being on antihypertensive treatment), diabetes mellitus (random blood glucose > 110 mg/dl or being on antidiabetic treatment), hypercholesterolemia (total cholesterol > 200 mg/dl), smoking and alcohol drinking history. It was suggested that the diagnosis of diseases, consisting of hypertension, diabetes mellitus and hypercholesterolemia, were based on the International Classification of Diseases, 10th Revision (ICD-10). In addition, previous research findings suggested that alcohol drinking, especially daily drinking in elderly people was as an increased risk factor of cognitive impairment, so that, frequent alcohol drinking was as an critical value in this study (Anttila et al., 2004; Zhou et al., 2014). Similarly, more than 25 pack-years of cigarettes calculated by multiplying the number of packs of cigarettes per day by the number of years of smoking was as an critical point in this study (Zhou et al., 2014; Ohara et al., 2015). Consequently, six subgroups based on quantities of VRF were in turn none-vascular risk factor (NVRF) (MCI = 24, controls = 16), One-VRF (MCI = 21, controls = 20), and Over-One-VRF (MCI = 22, controls = 25).

### Neuropsychological tests

All subjects underwent a clinical interview performed by an experienced neuropsychologist that involved in a demographic inventory, medical history, and neurological and mental status examinations. General cognitive functioning was evaluated by a mini-mental state examination (MMSE) and Mattis dementia rating scale (MDRS). In addition, a neuropsychological battery that consisted of AVLT-DR, Logical Memory Test-DR (LMT-DR), Rey-Osterrieth Complex Figure Test-DR (CFT-DR), Clock Drawing Test (CDT), Rey-Osterrieth Complex Figure Test (CFT), Digit Symbol Substitution Test (DSST), Digit Span Test (DST), Trail Making Test-A and -B (TMT-A and TMT-B), Verbal Fluency Test-Objects and -Animals (VFT-objects and VFT-animals), Similarity and Stroop Color and Word Tests A, B, and C was used to evaluate the functions of episodic memory (EM), visuospatial function (VF), information processing speed (IPS) and executive function (EF).

### ApoE genotyping

Genomic DNA from each subject was extracted from 250 μL EDTA-anticoagulated blood by using a DNA direct kit (Tiangen, China). To detect the rs7412 and rs429358 alleles, a polymerase chain reaction-based restriction fragment length polymorphism assay was employed, and the ApoE genotype was ultimately determined by the haplotype.

### MRI data acquisition

All of the subjects were scanned by a Siemens Verio 3.0-T scanner (Siemens, Erlangen, Germany) with a homogeneous birdcage head coil in order to reduce head movements. For one thing, high-resolution T1-weighted axial images covering the whole brain acquired by a 3D-magnetization prepared rapid gradient echo sequence as follows: repetition time (TR) = 1,900 ms, echo time (TE) = 2.48 ms, flip angle (FA) = 9°, acquisition matrix = 256 × 256, number of slices = 176, thickness = 1.0 mm, gap = 0 mm, and feld of view (FOV) = 250 × 250 mm^2^. For another, the resting-state functional scans covering 240 volumes were obtained with a gradient-recalled echoplanar imaging (GRE-EPI) sequence: TR = 2,000 ms, TE = 25 ms, FA = 90°, acquisition matrix = 64 × 64, number of slices = 36, thickness = 4.0 mm, gap = 0 mm, and FOV = 240 × 240 mm^2^.

### Image preprocessing

The fMRI data obtained from each subject were preprocessed by Data Processing Assistant for Resting-State fMRI (DPARSF) V2.1 (http://www.restfmri.net/forum/DPARSF) based on the Statistical Parametric Mapping 8 (SPM8) toolkit (www.fil.ion.ucl.ac.uk/spm) and MATLAB (The Math Works, Inc.; Natick, MA, USA) programs. The first 10 volumes of the scanning session were abandoned to allow for magnetization equilibration effects. Then, the remaining images were corrected for timing differences in acquisition among slices and head motion effects. No subjects performed a head motion > 3.0 mm of displacement or >3.0° of rotation during the scan. Next, the obtained images were spatially normalized into Montreal Neurological Institute echo-planar imaging template, resampled to 3 × 3 × 3 mm^3^ voxels, and smoothed with a Gaussian kernel of 6 × 6 × 6 mm^3^ (full width at half-maximum, FWHM). The nuisance signals including 24 head motion parameters, and global mean, white matter and cerebrospinal fluid signals were regressed out as covariates of no interest. Finally, The resulting data were band-pass filtered within the frequency range of 0.01 and 0.08 Hz to reduce the low-frequency drift and high frequency cardiac noise and physiological respiratory.

### Seed-based functional connectivity analysis

A spherical region of interest (ROI) (radius = 10 mm) as seeds for the connectivity analysis was centered at the given coordinates (−5, −49, 40) located in PCC (Wang et al., 2012). For each subject, a average time series for ROI was computed as the reference time course. Pearson cross-correlation analysis was then conducted between the average signal change in the PCC and the time series of whole-brain voxels. Next, a Fisher's z-transform (*z* = 0.5 × ln(1+r)/(1-r)) was used to improve the normality of the correlation coefficients. Finally, the individual DMN map was constructed.

### Statistical analysis

#### Demographic and neuropsychological data

The composite scores were applied to enhance statistical reliability by means of reducing random variability and eliminating the floor and ceiling effects (Wilson et al., 2010). The composite scores were obtained as the following steps: Firstly, the raw scores from some tests (including TMT-A, TMT-B, Stroop A, Stroop B, and Stroop C) for individuals were transformed into reciprocal for maintaining consistency with other tests. Secondly, the remaining and processed raw scores were transformed into z-scores with reference to the overall means and SD of all participants. Finally, the composite scores representing cognitive domain scores were calculated by averaging the z-scores of the individual tests as follows: EM score included the AVLT- DR, LMT-DR, and CFT-DR scores, VF score included the CDT and CFT scores, and IPS score included the DSST, TMT-A, Stroop A, and Stroop B scores, and EF score included the VFT-objects, VFT-animals, DST-backward, TMT-B, Stroop C, and Similarity scores.

Chi-square (χ^2^) tests (only applied in enumeration data: gender and APOE ε4), two-way analysis of variance (ANOVA) and two-way analysis of covariance (ANCOVA) were conducted in the comparison of the demographic data and neuropsychological performances between the six subgroups with statistically significant differences (*p* < 0.05). All statistical analyses were performed using SPSS 19.0 software (SPSS, Inc.; Chicago, IL, USA).

#### Group-level intrinsic connectivity analysis

Within the six subgroups, individual z-values were conducted to a one-sample *t*-test in a voxel-wise manner to identify the brain regions showing significant connectivity to PCC, and voxels with *p* < 0.05 and a minimum cluster size of 148 voxels were used to correct for multiple comparisons by Monte Carlo simulation (corrected *p* < 0.05). Two-way ANCOVA with state of VRF (i.e., NVRF, One-VRF, and Over-One-VRF) and disease [i.e., MCI and healthy control (HC)] was performed, with age, gender, years of education, and APOE genotype (ε4 carriers and non-carriers) as covariants. Statistical F-tests for the interaction effect of both factors were performed, and subsequent analyses were restricted to the DMN mask which was set at a combined threshold of *p* < 0.01 and a minimum cluster size of 24 voxels (corrected *p* < 0.05, determined by Monte Carlo simulation). In addition, a *post hoc* test related to interaction regions was applied in comparison between the subgroups.

#### Correlative analysis of functional connectivity with behavioral performance scores in interaction regions

To explore whether there is a linear relation between the significant functional connections and the neuropsychological performances, a multivariate regression analysis was performed to examine the potential relationship between abnormal z-values of functional connectivity and behavioral performance scores in HC and MCI subjects. The equation is refering to the related study from Xie et al. and add APOE ε4 as a new covariate in present study (Xie et al., 2012). Similarly, all statistical procedures utilized the SPSS 19.0 software (SPSS, Inc., Chicago, IL, USA).

## Results

### Demographic and neuropsychological performance

Demographic characteristics and neuropsychological performance for all subjects are shown in Table 1. There were no significant differences between subgroups in terms of age, gender, years of education and APOE genotype (ε4 carriers and non-carriers). In consideration of the main effect of disease, MCI subjects displayed significantly poor performances on general cognition (including MMSE and MDRS), EM, VF, information processing speed and executive function compared with HC subjects. Additionally, no significant differences in neuropsychological performance were observed in regard to the main effect of VRF and the interaction effect on Stroop B scores showed significant difference between subgroups.

**Table 1:** Demographic and neuropsychological data.

**Items**	**HC**			**MCI**			***p*****-values**		
	**NVRF (*n* = 16)**	**One-VRF (*n* = 20)**	**Over-one-VRF (*n* = 25)**	**NVRF (*n* = 24)**	**One-VRF (*n* = 21)**	**Over-one-VRF (*n* = 22)**	**Diagnosis**	**VRF**	**Interaction or χ^2^**
									
**DEMOGRAPHICS**									
Age (years)	66.25 ± 5.60	68.90 ± 5.40	68.12 ± 7.01	71.04 ± 8.17	67.81 ± 7.65	68.23 ± 7.12	0.312^b^	0.953^b^	0.146^b^
Education (years)	12.06 ± 2.81	13.35 ± 3.22	12.58 ± 2.76	12.17 ± 2.90	11.19 ± 3.08	11.70 ± 3.47	0.075^b^	0.970^b^	0.255^b^
Gender (male/female)	5/11	8/12	11/14	7/17	11/10	16/6	–	–	0.050^a^
APOE ε4 (YES/NO)	6/10	8/12	10/15	11/13	5/16	7/15	–	–	0.730^a^
**GENERAL COGNITION**									
MMSE	28.56 ± 0.89	28.95 ± 1.32	27.88 ± 1.54	25.96 ± 2.35	26.57 ± 3.31	26.64 ± 2.52	<0.001^*^	0.466	0.368
MDRS	138.38 ± 3.03	138.20 ± 3.16	137.72 ± 4.85	132.42 ± 6.68	131.95 ± 6.68	131.09 ± 8.09	<0.001^*^	0.542	0.659
**COMPOSITION Z SCORES OF EACH COGNITIVE DOMAIN**									
Episodic Memory	0.59 ± 0.54	0.74 ± 0.55	0.53 ± 0.45	−0.66 ± 0.67	−0.47 ± 0.68	−0.53 ± 0.69	<0.001^*^	0.442	0.803
AVLT-DR (raw score)	7.69 ± 2.36	7.85 ± 1.81	7.464 ± 1.91	2.50 ± 1.67	2.43 ± 1.50	2.50 ± 1.65	<0.001^*^	0.993	0.701
LMT-DR (raw score)	8.50 ± 2.48	8.33 ± 3.20	7.86 ± 2.13	4.06 ± 3.35	5.21 ± 3.64	5.52 ± 3.06	<0.001^*^	0.904	0.520
CFT-DR (raw score)	17.69 ± 6.96	20.95 ± 4.29	17.88 ± 4.75	12.04 ± 7.12	13.83 ± 6.35	11.86 ± 7.16	<0.001^*^	0.099	0.467
Visuospatial Function	0.35 ± 0.39	0.35 ± 0.39	0.19 ± 0.54	−0.50 ± 1.23	−0.06 ± 0.71	−0.19 ± 0.68	<0.001^*^	0.493	0.693
CDT (raw score)	9.19 ± 0.83	9.15 ± 0.99	8.60 ± 1.35	7.75 ± 1.57	8.19 ± 1.66	7.64 ± 2.22	<0.001^*^	0.133	0.987
CFT (raw score)	34.31 ± 1.78	34.40 ± 1.85	34.48 ± 1.66	31.50 ± 6.51	33.62 ± 2.46	33.91 ± 2.18	0.057	0.381	0.541
Information Processing Speed	0.40 ± 0.57	0.49 ± 0.79	0.49 ± 0.65	−0.31 ± 0.80	−0.48 ± 0.62	−0.50 ± 0.84	<0.001^*^	0.962	0.386
DSST (raw score)	43.63 ± 7.98	41.40 ± 9.84	38.88 ± 9.55	31.13 ± 10.36	29.48 ± 9.43	30.82 ± 11.50	<0.001^*^	0.469	0.506
TMT-A (raw score, second)	67.56 ± 12.76	64.10 ± 20.78	66.52 ± 17.82	89.58 ± 54.26	82.90 ± 22.61	80.14 ± 6.56	0.008^*^	0.799	0.950
Stroop A (raw score, second)	25.13 ± 3.16	25.80 ± 5.75	23.72 ± 3.98	31.46 ± 8.82	31.76 ± 7.63	34.36 ± 10.37	<0.001^*^	0.893	0.117
Stroop B (raw score, second)	38.44 ± 11.14	36.70 ± 8.49	35.08 ± 6.27	43.42 ± 10.89	48.86 ± 11.53	53.45 ± 17.40	<0.001^*^	0.684	0.022^*^
Executive Function	0.40 ± 0.39	0.46 ± 0.52	0.43 ± 0.64	−0.27 ± 0.63	−0.42 ± 0.57	−0.50 ± 0.58	<0.001^*^	0.620	0.296
VFT-objects (raw score)	26.88 ± 4.19	26.95 ± 5.45	26.84 ± 6.31	20.46 ± 6.77	20.38 ± 5.41	18.55 ± 7.39	<0.001^*^	0.826	0.624
VFT-animals (raw score)	26.63 ± 3.58	24.30 ± 5.79	21.60 ± 5.07	15.88 ± 4.85	17.05 ± 4.54	15.55 ± 4.70	<0.001^*^	0.068	0.941
DST-backward (raw score)	4.75 ± 1.00	5.05 ± 1.19	5.40 ± 1.91	4.54 ± 1.50	4.29 ± 1.42	4.50 ± 1.19	0.122	0.508	0.546
TMT-B (raw score, second)	157.56 ± 47.25	183.15 ± 50.51	167.60 ± 56.99	251.13 ± 144.26	248.14 ± 103.47	233.64 ± 64.96	<0.001^*^	0.406	0.928
Stroop C (raw score, second)	75.31 ± 23.14	73.95 ± 18.28	71.56 ± 15.37	88.46 ± 25.13	101.76 ± 29.65	108.73 ± 35.35	<0.001^*^	0.432	0.069
Similarity (raw score)	19.50 ± 2.25	20.55 ± 2.61	19.28 ± 3.20	17.79 ± 2.57	16.86 ± 4.17	16.64 ± 4.18	<0.001^*^	0.403	0.435

−*Values are presented as the mean ± standard deviation (SD).*

a *The p-value was obtained by χ^2^ test*.

b *The p-value was obtained by two-way ANOVA and two-way ANCOVA was applied in the other comparisons*.

* *Indicates a statistical difference between groups, p < 0.05*.

*HC, health control; MCI, mild cognitive impairment; VRF, vascular risk factor; NVRF, none vascular risk factor; MMSE, mini mental state examination; MDRS, Mattis Dementia Rating Scale; AVLT-DR, Auditory Verbal Learning Test-delayed recall; LMT-DR, Logical Memory Test-delayed recall; CFT, Rey-Osterrieth Complex Figure Test; CFT-DR, Rey-Osterrieth complex figure test-delayed recall; CDT, Clock Drawing Test; DSST, Digit Symbol Substitution Test; DST, Digit Span Test; VFT, Verbal Fluency Test; TMT-A and TMT-B, Trail Making Tests-A and B; Stroop A, B, and C, Stroop Color and Word Tests A, B, and C*.

### Functional connectivity analysis

#### Functional connectivity analysis-within group comparison

**Figure 1:**
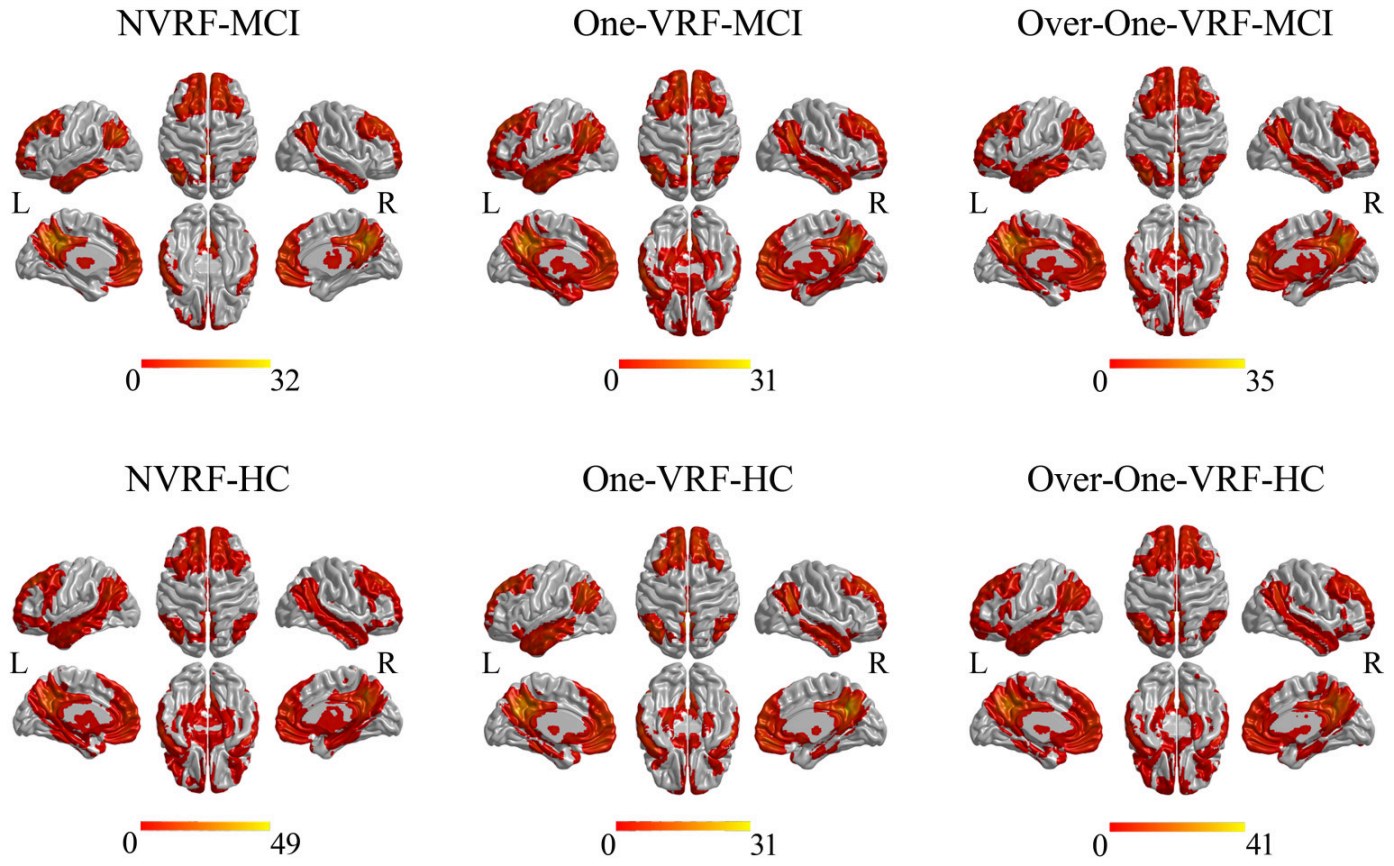
The DMN networks constructed by PCC as the seed region. The thresholds were set at *p* < 0.05 and a minimum cluster size of 148 voxels (corrected *p* < 0.05, determined by Monte Carlo simulation for multiple comparisons). L, left; R, right; VRF, vascular risk factor; NVRF, none-vascular risk factor; MCI, mild cognitive impairment; HC, healthy control.

Both in HC and MCI with or without VRF, the DMN networks showed similar functional connectivity patterns which mainly covered other subregions including ventromedial prefrontal cortex, lateral inferior parietal lobes and medial temporal lobes in line with previous studies (Leech and Sharp, 2014; Bai et al., 2016; Figure 1).

#### Functional connectivity analysis-between group comparison

**Figure 2:**
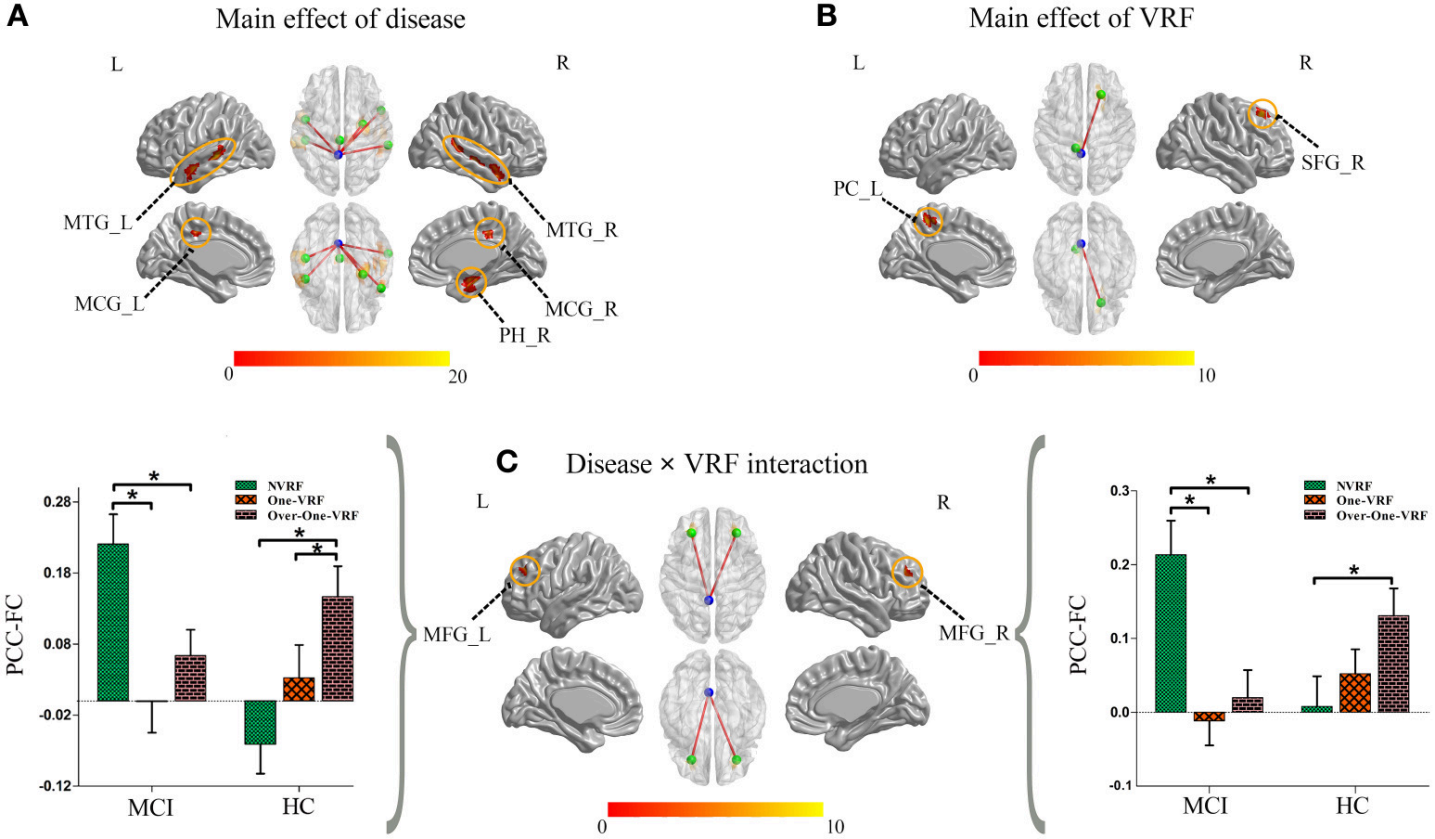
**(A)** The main effect of disease on the PCC-FC network. **(B)** The main effect of VRF on the PCC-FC network. **(C)** The interaction of disease × VRF on the PCC-FC network. Post hoc tests were also performed. The error bars represented standard error of the means of FC. ^*^P < 0.05. (The thresholds were set at *p* < 0.01 and a minimum cluster size of 24 voxels (corrected *p* < 0.05, determined by Monte Carlo simulation for multiple comparisons). L, left; R, right; VRF, vascular risk factor; NVRF, none-vascular risk factor; MCI, mild cognitive impairment; HC, healthy control; MTG, middle temporal gyrus; MCG, middle cingulate gyrus; PH, parahippocampal gyrus; PC, precuneus cortex; SFG, superior frontal gyrus; MFG, middle frontal gyrus; FC, functional connectivity; PCC, posterior cingulate cortex.

The main and interactive effects of disease and VRF were displayed in Table 2 and Figure 2. Regions related to the main effect of disease were observed in right parahippocampal gyrus and bilateral middle temporal gyrus and middle cingulate gyrus, while the main effect of VRF was detected in right superior frontal gyrus and left precuneus. Additionally, effects of disease × VRF interaction was primarily in bilateral dorsolateral prefrontal cortex (DLPFC) (i.e., middle frontal gyrus).

**Table 2:** Disease × VRF ANCOVA of PCC-FC.

**Brain region**	**BA**	**Peak MNI coordinates x, y, z (mm)**	**Peak *F*-value**	**Cluster size (mm^3^)**
**(1) MAIN EFFECTS OF DISEASE**				
R Parahippocampal Gyrus	36	27, −9, −24	18.24	3,861
B Middle Cingulate Gyrus	23	−3, −30, 42	11.04	783
R Middle Temporal Gyrus	21	51, 9, −27	12.50	675
R Middle Temporal Gyrus	21	60, −36, 9	9.96	1,161
L Middle Temporal Gyrus	21	−48, −3, −12	14.77	1,917
L Middle Temporal Gyrus	21	−48, −30, 0	19.05	1,512
**(2) MAIN EFFECTS OF VRF**				
R Superior Frontal Gyrus	8	21, 27, 51	7.39	1,161
L Precuneus	29	−12, −42, 54	8.37	918
**(3) DISEASE** × **VRF INTERACTION**				
R Middle Frontal Gyrus	46	30, 36, 33	7.94	918
L Middle Frontal Gyrus	46	−27, 36, 33	9.82	918

*The thresholds were set at a corrected P < 0.05, determined by Monte Carlo simulation for multiple comparisons. BA, Brodmann's area; MNI, Montreal Neurological Institute; R, right; L, left; B, bilateral*.

The *post hoc* test mainly revealed the intercomparison of functional connectivity between PCC and interactive regions (bilateral DLPFC) in all six subgroups (Figure 2). Intriguingly, MCI and HC subjects showed a similar pattern toward functional connectivity of PCC with bilateral DLPFC. In HC group with the increased load of VRF, PCC displayed more and more intensive connectivity to bilateral DLPFC. In other hand, MCI with One-VRF showed lower connectivity compared to NVRF subgroup whereas functional connectivity in Over-One-VRF rose again. In brief, normal elderly showed the gradually increased functional activity of DLPFC, while a fluctuant activation of DLPFC was displayed in MCI with the growing number of the VRF.

### Behavioral significance of PCC functional connectivity

**Figure 3:**
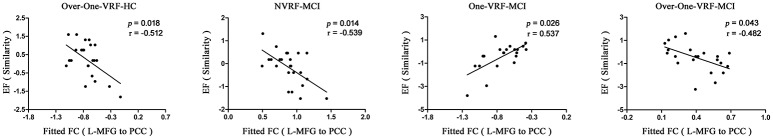
Fitted functional connectivity and behavioral significance. The raw scores of the tests for each subject were transformed to Z scores. L, left; R, right; VRF, vascular risk factor; NVRF, none-vascular risk factor; MCI, mild cognitive impairment; HC, healthy control; EF, executive function; FC, functional connectivity; PCC, posterior cingulate cortex; MFG, middle frontal gyrus.

**Figure 4:**
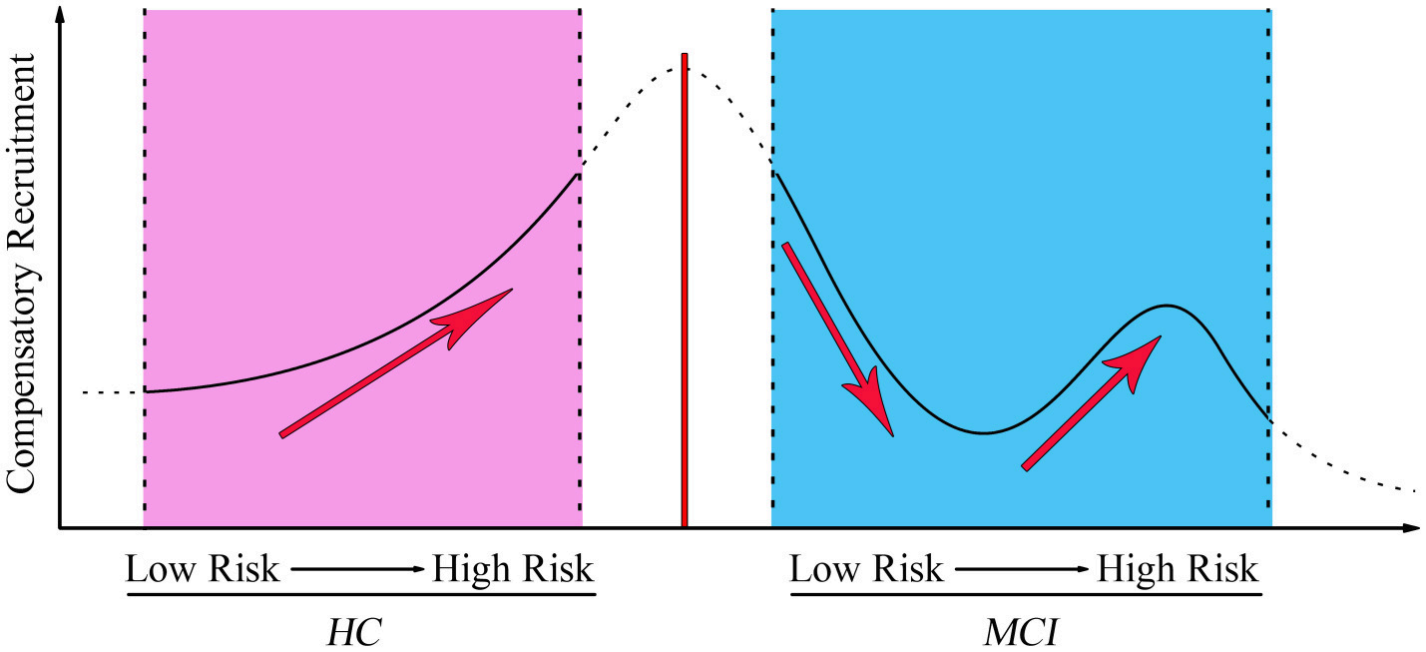
The fluctuation changes of PCC-FC with the left middle frontal gyrus along with vascular factors from low risk to high risk. MCI, mild cognitive impairment; HC, healthy control.

To investigate the behavioral significance of PCC functional connectivity with interactive regions (bilateral DLPFC), the significant results are described as follows by a multivariate regression analysis (Figure 3). We found an intriguing pattern about the relation between executive function (subdomain: Similarity) and altered function connectivity of PCC within the left DLPFC. Firstly, in Over-One-VRF-HC subgroup, the left DLPFC was significantly negatively correlated with performance on the Similarity (*r* = −0.512, *p* = 0.018). And then, the left DLPFC displayed significantly negative relation to the performance of the Similarity (*r* = −0.539, *p* = 0.014) in NVRF-MCI subgroup. When MCI with Over-One VRF, the alteration of PCC with the left DLPFC was positively correlated with the changes in Similarity scores (*r* = 0.537, *p* = 0.026). Furthermore, significantly negatively correlation between Similarity scores and increased functional connectivity was found in MCI subgroup with Over-One VRF (*r* = −0.482, *p* = 0.043). In summary, the left DLPFC further displayed significantly dynamic correlation with executive function as the variation of VRF loading. Initial level of compensation was observed in normal aging and NVRF MCI, while these compensatory neural processes were suppressed in One-VRF MCI and were subsequently re-aroused in Over-One-VRF MCI (Figure 4).

## Discussion

In this study, our main goal was to investigate underlying effects of VRFs on the DMN and to explore the behavioral correlations in MCI. Our results showed that the interactive effects of VRF on the DMN in MCI were primarily in the bilateral DLPFC, specifically in the left DLPFC. This was significantly correlated with executive function. Notably and intriguingly, the undulant alterations of the left DLPFC functional connectivity were observed along with quantity variations of VRF. We suggest that the dose-dependent effects of VRF on DLPFC were highlighted in MCI. The dynamic compensatory neural processes that fluctuated along with the variations of VRF loading could play key role in the progression of MCI.

### Altered DLPFC-PCC network related to AD progression due to vascular risks

The DLPFC is considered to be a vital element of the prefrontal cortex (PFC) subdivided into a series of separate, yet interconnecting areas. It is thought to be closely associated with the performance of the executive functions (Funahashi and Andreau, 2013; Brunoni and Vanderhasselt, 2014; Gerrits et al., 2015). The processes involved in the executive function include controls of attention, working memory, planning behavior, self-regulation, and cognitive flexibility. Each of these cognitive processes is based on various neural circuits (Tisserand and Jolles, 2003). The DLPFC is thought to send “top-down control signaling” to other cortical and subcortical areas in a way that efficiently supervises the integration and coordination of multiple neural activities (Funahashi and Andreau, 2013). The left DLPFC had a tendency to be more associated with the cortical regions so-called “flexible hubs” in the DMN. This was evidenced by task-related fMRI analyses (Raichle and Snyder, 2007; Cole et al., 2013; Hakun et al., 2015).

The results from our study suggested that bilateral DLPFC (particularly in the left DLPFC) acted as interactive regions and showed significant correlations with executive function due to the effects of VRF in MCI. The interrelation between the DLPFC and the executive function was substantiated by AD-associated research. The increased DLPFC connectivity in AD patients when compared to both MCI and normal elderly was significantly correlated with executive function (Agosta et al., 2012). The early-onset AD had decreased function connectivity in the DLPFC relative to the controls. The reverse pattern was found in late-onset AD, and the DLPFC connectivity had a correlation with executive performances (Gour et al., 2014). There are few studies that observe the altered DLPFC networks as an effect of VRF in AD. There were some studies that were devoted to exploring the effects of single VRF on the DMN in AD individuals (Chen et al., 2014, 2016; Cui et al., 2015; Son et al., 2015), rather than focusing on the aggregation effect of multiple VRFs. The aberrant pattern of the DMN has been extensively studied in AD-spectrum subjects with various VRFs (Chen et al., 2014; Son et al., 2015), however, the agreement has not been applied to the specific cortical regions that are more vulnerable to the VRFs burden.

Our study suggested that the altered DLPFC network was represented as the specific site, due to the VRFs burden in the progression of AD. The potential mechanism and neural underpinning remain poorly defined. Subjects with high levels of VRFs have thinner cortex that is confined to frontotemporal regions independent of Aβ deposition (Villeneuve et al., 2014). A previous study proposed that selective regional pyramidal cell volumes were reduced by 30–40% compared to controls, rather than the neuronal density in the DLPFC of AD subjects (Foster et al., 2014). Metabolism-related research has emphasized the oxidative stress plays a crucial role in the metabolic disturbance due to the vascular risks and the initiation of the pathological process relevant to Aβ deposition in AD (Zhu et al., 2007). In high levels of VRFs burden, the prefrontal N-acetylaspartate/creatine (NAA/Cr) ratios were markedly lower than the low-risk individuals. This was associated with executive function via a magnetic resonance spectroscopy (MRS) study (Sun et al., 2014). The mitochondrial morphological abnormalities that were attributed to oxidative stress were identified in the DLPFC, where mRNA of mitochondrial proteins was expressed intensively, as vital part of the AD progression (Ansari and Scheff, 2010). The ultrastructural evidences in rhesus monkeys helped to illuminate this process. They indicated that mitochondrial changes in the DLPFC could play a critical role in cognitive decline (Hara et al., 2014; Morozov et al., 2017). The potential mechanism of DMN remodeling that results from the VRFs could be associated with metabolic dysfunction and structural basis in the DLPFC. AD is a complex and heterogeneous disease that affects discrete brain regions with defined characteristics for certain areas and pathways, which deserves intensive investigation. The significant DLPFC network that was correlated with executive function could provide an imaging biomarker for AD by carrying VRFs to identify and monitor the disease development.

### Dynamic compensatory neural processes fluctuated with variation of VRF loading

Rs-fMRI studies that address the brain network alterations caused by the number of the VRF have been scarce. In the current study, we observed the increased functional connectivity in the left DLPFC that was correlated with executive function. This was done by the variation of VRF loading in cognitively normal individuals in contrast to the undulant pattern in MCI. There are two plausible explanations for the increased functional connectivity (Tisserand and Jolles, 2003; Park and Reuter-Lorenz, 2009; Cox et al., 2015). It could reflect an attempt to recruit the additional resources to supplement the function of a disrupted network. This is referred to as the “prefrontal effort hypothesis” or “partial compensation hypothesis.” It could also be because such differences manifest an inability to shut down unessential activity. The “inhibitory hypothesis” or “decreased neural efficiency” states that this activity will not cease even when it is potentially detrimental to the task performance via the anterior corpus callosum.

We assumed that the increased left DLPFC-PCC negatively correlated with the executive function as a compensatory mechanism that would offset the detrimental impact on cognitive performance. As with the previous study, the increased PCC functional connectivity was primarily found in the left frontal-parietal cortical regions. This was thought to be impaired later and could be preferential compensation for the disrupted brain networks earlier (Zhang et al., 2010). The evidence has suggested that the additional activity in the DMN could refer to the amyloidogenic pathway where the endogenous neuronal activity promotes Aβ production and transitions to the interstitial fluid (Bero et al., 2011; Ovsepian and O'Leary, 2016). Yamamoto et al. directly confirmed that the excessive neuronal activity was remarkably associated with an increased release of Aβ production and amyloid plaque formation in *in vivo* AD models (Yamamoto et al., 2015). The increased DMN activity could respond to a neuropathological processing that leads to amyloid plaques burden. This subsequently contributed to the gradually decline of functional connectivity in those regions.

The normal elderly group gradually increased the functional activity of DLPFC with the growing number of the VRF. A task-related fMRI study indicated that higher vascular risk including systolic blood pressure and body mass index was significantly associated with increased activation in PCC and DLPFC, parietal and temporal regions in cognitively intact older adults. These relationship was evident even in normal ranges of systolic blood pressure and body mass index (Braskie et al., 2010). This result also was in keeping with another neuroimaging evidence that found increased fMRI signal in medial temporal lobe in the elderly with increased vascular risk (Bangen et al., 2009). This increased activation may reflect a compensatory mechanism by increasing recruitment of cognitive resources to maintain performance. Individuals in the hypertension group with APOE ε4 allele, specifically in the high vascular risk group, showed significantly greater levels of Aβ deposition than individuals with a single VRF or none at all. This research demonstrated a dose-response impact of the increase in pulse pressure on elevated brain Aβ load (Rodrigue et al., 2013). A previous study highlighted the bivariate model to present the correlation between the Framingham Coronary Risk Profile score (FCRP) and global PIB index in the elderly and non-demented sample groups (Reed et al., 2012). From the perspective of disease incidence, the risk of conversion to AD increased with the number of the VRFs (Luchsinger et al., 2005). These evidences suggested a straight-line relationship between aggregate vascular risks and cerebral amyloid burden and AD incidence in the normal elderly. The MCI individual had a compensation pattern that manifested differentially. The initial level of compensation was observed in the NVRF subgroup. These compensatory neural processes were suppressed in the One-VRF subgroup and were subsequently re-aroused in the Over-One-VRF subgroup. The undulant compensation curve was distinct from the “inverse U-shaped curve” of compensatory enhancement of hippocampal FC to the frontal cortices. This suggested that the functional connectivity related to the recruitment compensatory would gradually decline with the depletion of the compensatory mechanisms (Jennings, 2003; Ye et al., 2017). These findings highlighted a more sophisticated pattern of neural compensation in mild stages of AD. This was predominate in the left DLPFC correlated with executive function, occurring along with the increase of VRFs burden. To our knowledge, dynamic compensatory neural processes fluctuated with variation of VRF loading have not been reported and the underlying mechanism of the undulant compensation curve is still not fully understood.

There were several limitations in our study. This study was performed on a relatively small sample size and we look forward to expanding the sample size to validate our results in future studies. In our study, the MCI participants mainly refer to aMCI, which is a transitional stage between normal aging and AD and is easier to convert to AD compared with other MCI subtypes (Sperling et al., 2011). What about the alterations of VRF-related DMN in non-aMCI participants need to be further explored. It was a cross-sectional study and it was impossible to dynamically follow up the brain network changes. We used the number of carried VRF as the gradational boundary. Previous studies sued the Cox proportional hazard models that correspond to FCRP in order to assess the severity of the VRFs (Jefferson et al., 2015). The FCRP was intended to forecast the incidence risk of stroke within the next decade. It is unknown this would agree with the VRF study for cognitive impairment. It is necessary to create a new vascular risk index for AD. There were different effects proportions of the VRFs to AD. In this study, we preliminarily setup the various number of the VRF and made refinements for each VRF. The aforementioned interpretations and results should be treated with caution.

## Conclusion

As a result, the current study could put forward the view that the dose-dependent effects of VRF on DLPFC may be a key role in identifying and monitoring the conversion to AD due to the neurovascular dysfunction. In addition, it is plausible that the dynamic neural processes might refer to compensatory neural recruitment fluctuated with variation of VRF loading. Identifying links between the dynamic compensation and vascular burden would be of of great significance to understand potential neural substrates in AD-associated progression.

## Author contributions

Conceived and designed the experiments: FB and HC. Performed the experiments: FS and QY. Analyzed the data: ZW. Contributed materials/analysis tools: HS.

### Conflict of interest statement

The authors declare that the research was conducted in the absence of any commercial or financial relationships that could be construed as a potential conflict of interest.
